# Transcriptomic and metabolomic analyses reveal that melatonin promotes melon root development under copper stress by inhibiting jasmonic acid biosynthesis

**DOI:** 10.1038/s41438-020-0293-5

**Published:** 2020-06-01

**Authors:** Zhicheng Hu, Qiushi Fu, Jing Zheng, Aiai Zhang, Huaisong Wang

**Affiliations:** Institute of Vegetables and Flowers, Chinese Academy of Agricultural Sciences, 100081 Beijing, China

**Keywords:** Metabolomics, RNA sequencing, Abiotic

## Abstract

Melatonin has been shown to alleviate the effects of abiotic stress and to regulate plant development. Copper, a common heavy metal and soil pollutant, can suppress plant growth and development. In this work, we explored the protective effects of exogenous melatonin on lateral root formation in response to copper stress using melon seeds subjected to three germination treatments: CK1 (control), CK2 (300 μmol/L CuSO_4_), and MT3 (300 μmol/L melatonin + 300 μmol/L CuSO_4_). Melatonin pretreatment increased the antioxidant enzyme activities and root vigor, and decreased the proline and malondialdehyde (MDA) contents in the roots of copper-stressed melon seedlings. We then used transcriptomic and metabolomic analyses to explore the mechanisms by which exogenous melatonin protects against copper stress. There were 70 significant differentially expressed genes (DEGs) (28 upregulated, 42 downregulated) and 318 significantly differentially expressed metabolites (DEMs) (168 upregulated, 150 downregulated) between the MT3 and CK2 treatments. Melatonin pretreatment altered the expression of genes related to redox and cell wall formation processes. In addition, we found that members of the AP2/ERF, BBR/BPC, GRAS, and HD-ZIP transcription factor families may have vital roles in lateral root development. Melatonin also increased the level of Glutathione (GSH), which chelates excess Cu^2+^. The combined transcriptomic and metabolomic analysis revealed DEGs and DEMs involved in jasmonic acid (JA) biosynthesis, including four lipoxygenase-related genes and two metabolites (linoleic acid and lecithin) related to melatonin’s alleviation effect on copper toxicity. This research elucidated the molecular mechanisms of melatonin’s protective effects in copper-stressed melon.

## Introduction

Melatonin (*N*-acetyl-5-methoxytryptamine) is an indoleamine ubiquitous in the plant kingdom^1^, and a modulator of multiple developmental processes and stresses^2,3^. Various types of research have demonstrated that melatonin can alleviate the effects of biotic and abiotic stresses, such as fungal, salt, and heavy metal stresses^4–6^. For example, Yin et al.^4^ found that apple trees pretreated with melatonin were more resistant to apple blotch than were control trees. The endogenous melatonin levels of *Arabidopsis* serotonin *N-acetyltransferase* knockout mutants were markedly lower than those of controls, resulting in susceptibility to an avirulent pathogen^7^. Under saline conditions, melatonin pretreatment significantly alleviated the inhibition of *Malus hupehensis* growth and may have reduced oxidative damage by enhancing antioxidative enzyme activities or eliminating H_2_O_2_^5^. Similarly, exogenous melatonin could alleviate the toxicity of cadmium stress in *Ulva* sp., a green macroalga^8^.

Melon (*Cucumis melo* L.) belongs to the Cucurbitaceae family and is widely planted throughout the world. Heavy metals, especially copper, have increasingly infiltrated both soil and water, becoming one of the most serious current environmental problems^6^. At low concentrations, copper is an essential microelement for plants^9^, whereas at high concentrations, it has physiological and biochemical toxicity effects on plants, as shown by increased generation of reactive oxygen species (ROS), disruption of protein structure, inactivation of enzymes, and so on^10^. In copper-stressed *Arabidopsis thaliana*, root and shoot lengths and chlorophyll content levels decreased, whereas the anthocyanin content increased^11^. High copper concentrations inhibited root and stem growth in *Arabidopsis* and reduced cell elongation, division, and expansion^12^. In addition, Cu^2+^ significantly induced protein oxidation and inhibited root growth in *Panax ginseng*^13^. Some research has suggested that the application of exogenous melatonin was able to protect plants from copper stress toxicity. Exogenous melatonin increased the tolerance of red cabbage seedlings to copper stress by decreasing lipid peroxidation, thus promoting seed germination and seedling growth^6^. Melatonin diminished the oxidative stress and proline accumulation caused by CuSO_4_, indicating that melatonin had a protective effect on canola plants under excessive copper^14^.

Roots not only are essential organs that absorb water and minerals from the soil, thus enabling plant growth and development, but also, importantly, are involved in the perception of stress signals^15,16^. Therefore, roots have a key role in the acclimation of plants to adverse conditions. Lateral roots are an important part of root systems, vastly increasing their surface area and mechanical strength^17^. Some studies have reported inhibitory effects of excessive copper on root development in different plant species, including *Arabidopsis*^12^, *Panax ginseng*^13^, radish^18^, citrus^19^, and sorghum^20^. The application of exogenous melatonin can promote root development, especially lateral root development. Melatonin-overexpressing transgenic rice seedlings showed more root formation and chlorophyll synthesis than did wild-type seedlings under cold stress^21^. In addition, application of melatonin to lupin promoted both morphogenesis of root primordia and the number of adventitious roots and lateral roots, as observed after indole-3-acetic acid (IAA) application^22^. Moreover, at low concentrations, exogenous melatonin promoted root growth of in vitro shoot tip explants of sweet cherry rootstocks, again with an effect similar to that of IAA^23^.

High copper levels are toxic to plants. Copper has increasingly infiltrated soil and water, seriously affecting plant growth and development^6,10^. Melatonin can increase plant tolerance to abiotic stress by decreasing oxidative damage and promoting root development. However, few studies have focused on the protective effects of exogenous melatonin on melon root formation under copper stress. In addition, the exact protective mechanisms elicited by exogenous melatonin remain unclear. In this work, we measured the root morphology and physiological traits, including antioxidant enzyme activities, MDA content, proline content, and root vigor, of plants subjected to eight treatments, and we ultimately selected seeds pretreated with 100 μmol/L melatonin from among six melatonin treatments to explore the mechanism by which melatonin protects melon roots from copper stress. We then combined transcriptomic and metabolomic analyses to compare the gene expression and metabolomic profiles between melatonin pretreatment and non-pretreatment groups under copper stress. The aim of this work was to identify the mechanisms of the protective effects of exogenous melatonin on the roots of melon seedlings under copper stress, which may be useful for the development of effective control measures against copper stress.

## Results

### Melatonin promoted melon root development under copper stress

**Fig. 1:**
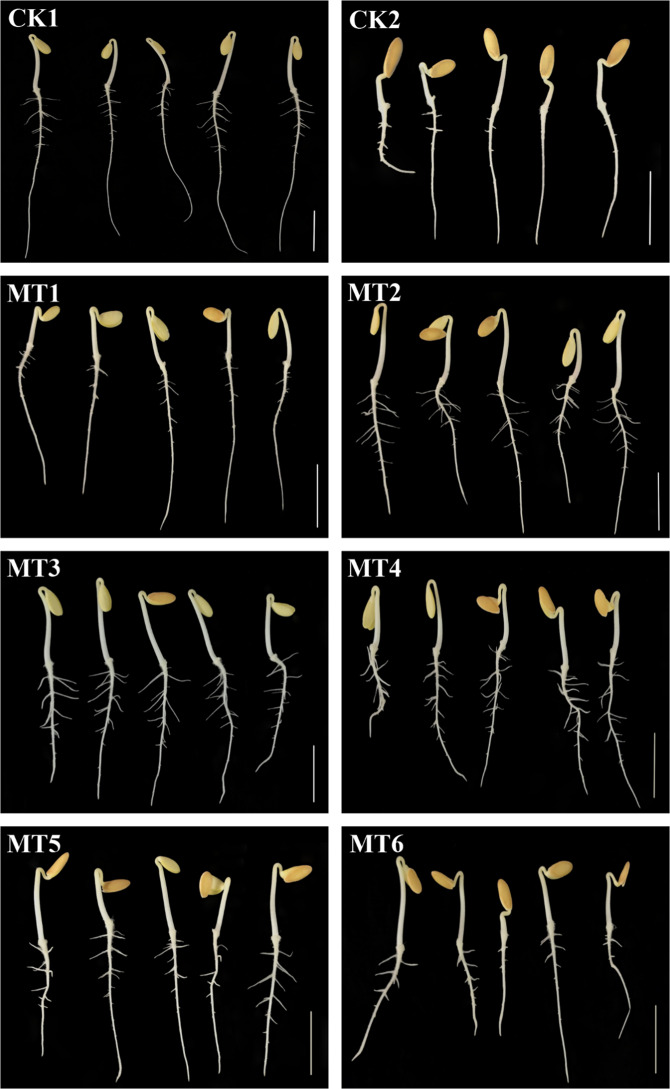
Effects of melatonin on melon root development under CuSO_4_ stress. CK1 non-stressed seeds pretreated with water, CK2 300 μmol/L CuSO_4_-stressed seeds pretreated with water, MT1–MT6 300 μmol/L CuSO_4_-stressed seeds pretreated with 10, 50, 100, 300, 500, or 800 μmol/L melatonin, respectively. Scale bar = 2 cm

The root morphology of melon seedlings under different treatments showed obvious differences. Untreated control (CK1) seedlings had long primary roots and many lateral roots. In comparison, the primary roots of copper-stressed (CK2) seedlings were shorter, with fewer lateral roots (Fig. 1). With respect to the CK2 seedlings, their root length, root surface area, and number of root tips decreased by 62%, 52%, and 70%, respectively, whereas the root average diameter compared with that of the CK1 seedlings increased by 40% (Table 1). Melatonin pretreatment promoted the development of melon roots (Fig. 1). In the melatonin pretreatment groups, the root surface area and root length increased significantly, especially between seedlings pretreated with 100 μmol/L melatonin (MT3) compared with the CK2 seedlings. The root surface area and root length of the MT3 seedlings increased by 183% and 168%, respectively. Excluding 10 μmol/L melatonin (MT1) seedlings, compared with that of the CK2 seedlings, the root volume of melatonin-pretreated seedlings significantly increased by 200%, 250%, 150%, 150%, and 150% under pretreatment with 50, 100, 300, 500, and 800 μmol/L melatonin, respectively, corresponding to the MT2–MT6 treatments. The average root diameter of the MT1–MT6 seedlings increased by 10%, 6%, 18%, 6%, 16%, and 14%, respectively, relative to that of the CK2 seedlings. The number of root tips number of the MT2–MT6 seedlings increased by 62%, 54%, 70%, 28%, and 13%, respectively, whereas that of the MT1 seedlings decreased by 5%, compared with that of the CK2 seedlings (Table 1). The MT1–MT6 seedlings revealed that melatonin pretreatment, especially the MT3 treatment, significantly promoted root development under CuSO_4_ stress.

**Table 1:** The effect of melatonin on melon root morphology under copper stress

Treatment	Root length/cm	Root surface area/cm^2^	Root volume/cm^3^	Root average diameter/mm	Root tip number
CK1	28.11 ± 6.15 a	3.09 ± 0.60 a	0.04 ± 0.01 b	0.35 ± 0.03 c	77.00 ± 13.78 a
CK2	10.68 ± 1.49 b	1.49 ± 0.19 c	0.02 ± 0.00 b	0.49 ± 0.06 b	23.40 ± 7.70 c
MT1	15.00 ± 3.90 b	2.35 ± 0.65 b	0.04 ± 0.01 b	0.54 ± 0.02 a	22.20 ± 2.68 c
MT2	27.43 ± 11.84 a	3.82 ± 1.80 a	0.06 ± 0.04 a	0.52 ± 0.82 a	38.00 ± 5.48 b
MT3	28.67 ± 9.15 a	4.21 ± 1.15 a	0.07 ± 0.03 a	0.58 ± 0.08 a	36.00 ± 7.18 b
MT4	27.41 ± 10.18 a	3.57 ± 1.20 a	0.05 ± 0.02 a	0.52 ± 0.07 a	39.80 ± 8.50 b
MT5	15.89 ± 5.57 b	2.73 ± 0.36 b	0.05 ± 0.01 a	0.57 ± 0.06 a	30.00 ± 7.48 bc
MT6	17.52 ± 3.94 b	2.84 ± 0.27 b	0.05 ± 0.01 a	0.56 ± 0.08 a	26.40 ± 8.44 c

Different letters within the same column indicate significant differences (*p* < 0.05, least significant difference test).

*CK1* non-stressed seeds pretreated with water, *CK2* 300 μmol/L CuSO_4_-stressed seeds pretreated with water, *MT1–MT6* 300 μmol/L CuSO_4_-stressed seeds pretreated with 10, 50, 100, 300, 500, or 800 μmol/L melatonin, respectively.

### Melatonin improved melon root resistance to excess copper

**Fig. 2:**
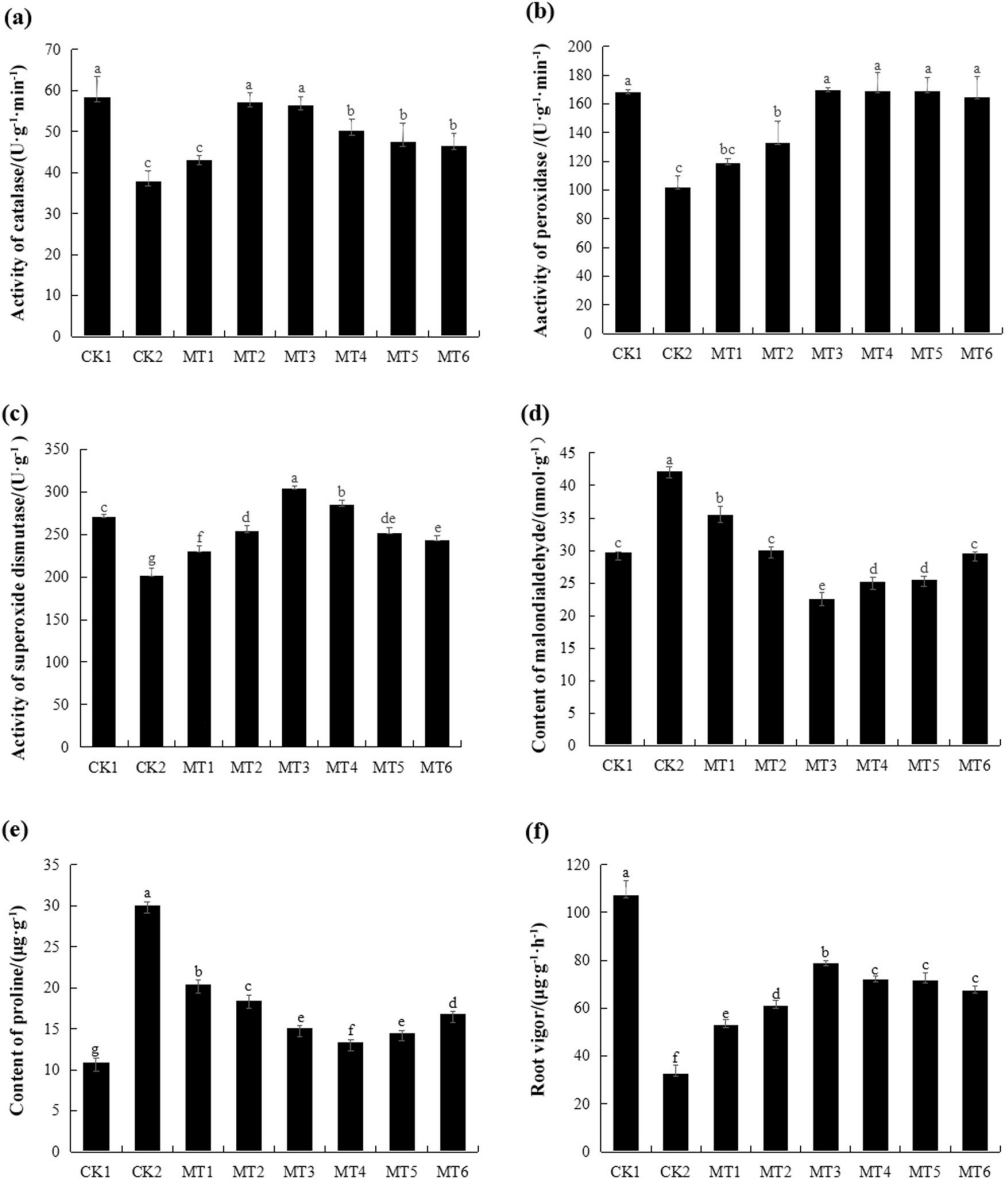
Effects of melatonin on physiologic indicators in CuSO_4_-stressed melon. **a** Effect of melatonin on the activity of catalase (CAT) in melon. **b** Effect of melatonin on the activity of peroxidase (POD) in melon. **c** Effect of melatonin on the activity of superoxide dismutase (SOD) in melon. **d** Effect of melatonin on the content of malondialdehyde (MDA) in melon. **e** Effect of melatonin on the content of proline in melon. **f** Effect of melatonin on the root vigor in melon. CK1 non-stressed seeds pretreated with water, CK2 300 μmol/L CuSO_4_-stressed seeds pretreated with water, MT1–MT6 300 μmol/L CuSO_4_-stressed seeds pretreated with 10, 50, 100, 300, 500, or 800 μmol/L melatonin, respectively

The activities of the antioxidant enzymes catalase (CAT), peroxidase (POD), and superoxide dismutase (SOD) significantly differed among the treatments. Compared with that in the CK1 seedlings, CAT, POD, and SOD activities in the CK2 seedlings decreased by 35%, 39%, and 26%, respectively. Under melatonin pretreatment, the activities of antioxidant enzymes were elevated compared with those in the CK2 seedlings: the CAT activity of the MT1–MT6 seedlings increased by 13.82%, 50.88%, 49.12%, 32.65%, 25.29%, and 23.24%, respectively, the POD activity of the MT1–MT6 seedlings increased by 16.17%, 30.25%, 66.13%, 65.21%, 65.69%, and 61.46%, respectively, and the SOD activity of the MT1–MT6 seedlings increased by 14%, 26%, 51%, 41%, 25%, and 21%, respectively (Fig. 2a–c).

The MDA content of the CK2 seedlings increased by 40% compared with that of the CK1 seedlings. In the MT1–MT6 seedlings, the MDA content decreased by 16.17%, 29.17%, 46.67%, 40.64%, 39.58%, and 30.10%, respectively, compared to that in the CK2 seedlings (Fig. 2d).

Compared with that in the CK1 seedlings, the proline content in the CK2 seedlings increased by 80%. Melatonin pretreatment decreased the proline content by 32.21%, 38.68%, 49.81%, 55.68%, 51.73%, and 44.07% in the MT1–MT6 seedlings, respectively, compared with that in the CK2 seedlings (Fig. 2e).

The root vigor of the CK2 seedlings was reduced by 69% relative to that of the CK1 seedlings and increased by 61.08%, 86.10%, 140.13%, 119.79%, 118.87%, and 105.29% in the MT1–MT6 seedlings, respectively, relative to that in the CK2 seedlings (Fig. 2f).

These results suggest that melatonin had a positive effect on melon root development under copper stress, with 100 μmol/L melatonin pretreatment displaying optimal effects.

Melatonin partly alleviated copper stress, with the best response detected for the 100 μmol/L (MT3) melatonin pretreatment. Therefore, we selected the CK1, CK2, and MT3 treatments for transcriptomic and metabolomic analyses to identify candidate genes and metabolites involved in the alleviation of copper toxicity in melon by melatonin.

### DEGs and DEMs induced by melatonin

#### Analysis of DEGs under melatonin pretreatment in response to copper stress

Three cDNA libraries were obtained by sequencing RNA extracted from the roots of CK1, CK2, and MT3 seedlings. Each library consisted of more than 6.3 Gb of clean data. More than 94.4% of the clean data had scores greater than Q30 in each library. In total, 90.64 to 92.31% of clean reads were mapped to the reference genome (Table 2).

**Table 2:** Summary of sequence data

Sample name	Clean reads	Clean bases	Mapped reads	Error rate (%)	Q30 (%)	GC content (%)
CK1	21,193,701	6,349,936,614	41,738,693 (92.31%)	0.1	94.47	42.48
CK2	22,601,871	6,757,021,369	38,414,231 (90.64%)	0.1	95.23	42.94
MT3	22,117,437	6,621,011,786	40,581,838 (91.73%)	0.1	94.74	42.76

*CK1* non-stressed seeds pretreated with water, *CK2* 300 μmol/L CuSO_4_-stressed seeds pretreated with water, *MT3* 300 μmol/L CuSO_4_-stressed seeds pretreated with 100 μmol/L melatonin.

**Fig. 3:**
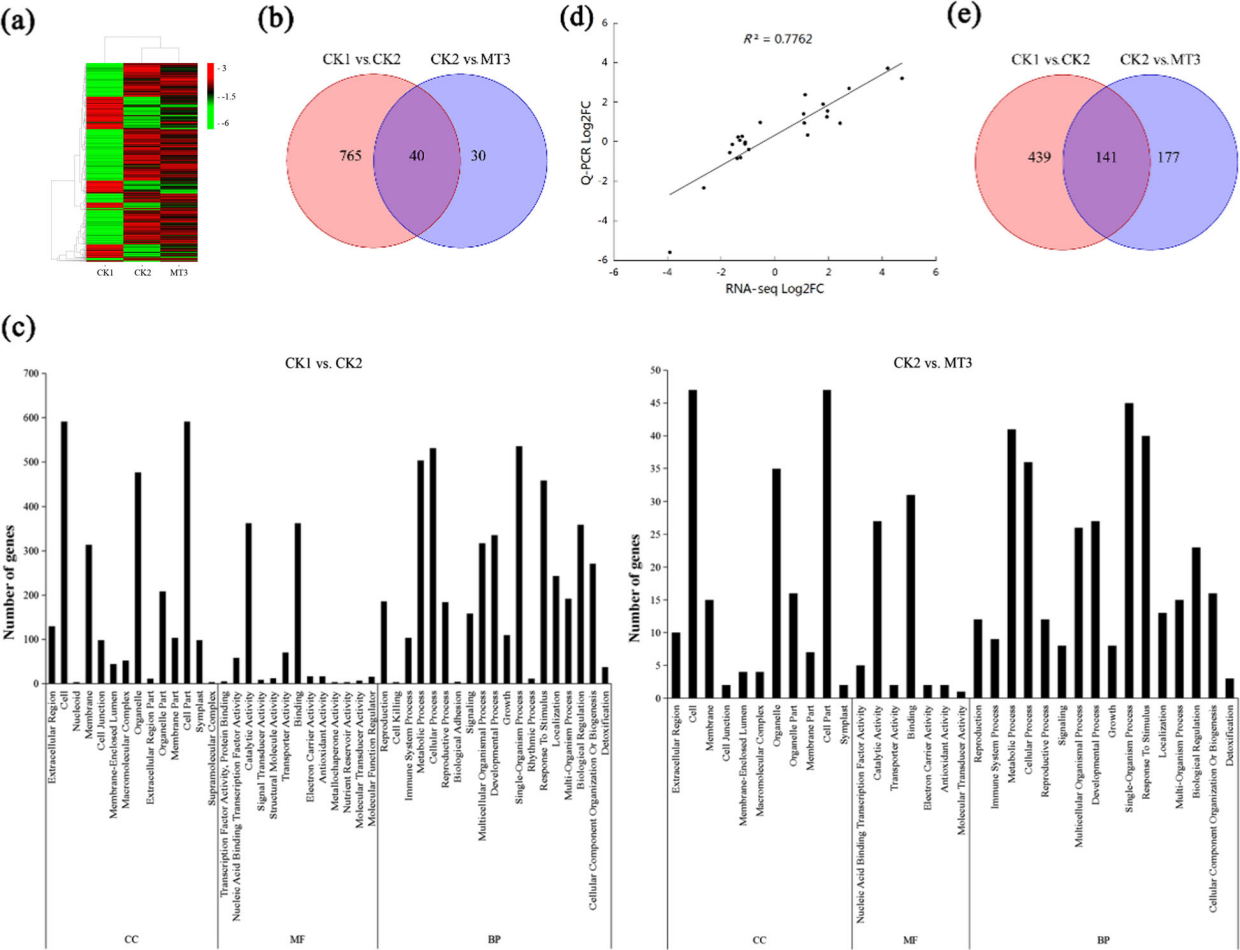
The differentially expressed genes (DEGs) and metabolites (DEMs) induced by melatonin. **a** Hierarchical clustering of DEGs induced by melatonin. **b** Venn diagram of DEGs. **c** Summary of Gene Ontology (GO) categories of the DEGs. **d** Relationship between RNA-seq and quantitative real-time PCR (Q-PCR) expression data (log_2_ fold change) (*R*^2^ = 0.7762). **e** Venn diagram of DEMs. CK1 non-stressed seeds pretreated with water, CK2 300 μmol/L CuSO_4_-stressed seeds pretreated with water, MT3 300 μmol/L CuSO_4_-stressed seeds pretreated with 100 μmol/L melatonin

On the basis of the sequencing of the three libraries, using a fold change ≥1.5 and a false discovery rate (FDR) < 0.05 as thresholds, DEGs from each two-treatment comparison (i.e., CK1 vs. CK2, CK1 vs. MT3, CK2 vs. MT3) were obtained. On the basis of the three treatments’ log_10_ (FPKM) values, a hierarchical cluster analysis of the DEGs was used to observe the overall expression pattern of the genes; green and red bands were used to represent low and high gene expression levels, respectively (Fig. 3a).

Figure 3b shows the differences in DEGs among the three treatments. We detected 805 DEGs (486 genes upregulated and 319 genes downregulated relative to those in CK1) associated with copper stress between the CK1 and CK2 melon root libraries. Furthermore, a total of 70 DEGs (28 genes upregulated and 42 genes downregulated relative to those in the CK2) were detected between the CK2 and MT3 libraries, which were thus associated with the response to exogenous melatonin and the alleviation of copper toxicity by melatonin (Supplementary Table S1). These genes that responded to exogenous melatonin were considered important candidates for further investigation.

The functions of the DEGs were classified according to Gene Ontology (GO) classifications. In total, 675 DEGs between CK1 and CK2 had GO annotations, most of which were enriched in the cell part, catalytic activity, single-organism process, organelle, and other functional categories. Between CK2 and MT3, there were 52 GO-annotated DEGs, which were mainly categorized into the cell part, binding, single-organism process, metabolic process, and other functional categories (Fig. 3c; Supplementary Table S1).

We also conducted a Kyoto Encyclopedia of Genes and Genomes (KEGG) enrichment analysis of the DEGs to identify the main pathways active in the melatonin-induced melon lateral root development process under copper stress. Between the CK1 and CK2 libraries, 159 DEGs were assigned to 78 KEGG pathways; phenylpropanoid biosynthesis (ko00940), with 28 genes (23 genes upregulated and 5 genes downregulated in CK2), and starch and sucrose metabolism (ko00500), with 17 genes (14 genes upregulated and 3 genes downregulated in CK2), were the major enriched pathways among the DEGs. Between CK2 and MT3, 11 DEGs were assigned to eight KEGG pathways; linoleic acid metabolism (ko00591), with four genes downregulated in the MT3, and endocytosis (ko04144), with three genes upregulated in the MT3, were the pathways most represented among these DEGs (Supplementary Tables S1 and S2).

To confirm the RNA-seq data, we assessed the expression of 12 randomly selected genes using quantitative real-time PCR (Q-PCR). As shown in Fig. 3d, the Q-PCR data were consistent with the RNA-seq data, and a significant positive correlation (*R*^2^ = 0.7762) supports the reliability of the RNA-seq data.

#### DEM analysis of melatonin pretreatment in response to copper stress

We conducted a metabolite profiling analysis of melon root samples (from CK1, CK2, and MT3 seedlings) to assess the overall metabolic effect of melatonin pretreatment on the roots of melon seedlings under copper stress. A total of 580 metabolites that significantly differed between the CK1 and CK2 treatments were detected, with 288 upregulated and 292 downregulated relative to those in the CK1 treatment. The levels of hydroxyhydroquinone, loquatoside, lucuminamide, and galactosylceramide, among others, increased by at least twofold, whereas the levels of 4-(beta-d-ribofuranosyl) aniline 5′-phosphate, 4′-hydroxytrazodone, and uridine 2′,3′-cyclic phosphate, among others, decreased by more than 60% under copper stress. Between the CK2 and MT3 treatments, 318 DEMs were detected, with 168 upregulated and 150 downregulated relative to those in the CK2 treatment. Val-Phe, l-mannomethylose and dolichyl diphosphate exhibited relatively high levels under melatonin pretreatment, whereas uridine, raffinose, and hexadecanedioic acid exhibited relatively low levels (Fig. 3e; Supplementary Table S3).

To identify the main pathways involved in the roots of melon seedlings under copper stress that responded to melatonin, we mapped the differentially expressed metabolites to KEGG biological pathways. As summarized in Supplementary Table S4, 52 significantly differentially expressed metabolites between the CK1 and CK2 treatments were assigned to 67 KEGG pathways, including metabolic pathway (ko01100), biosynthesis of secondary metabolites (ko01110), biosynthesis of amino acids (ko01230), and carbon metabolism (ko01200), among others. Between the CK2 and MT3 treatments, 30 significantly differentially expressed metabolites were enriched in 44 KEGG pathways, including metabolic pathway (ko01100), biosynthesis of secondary metabolites (ko01110), pyrimidine metabolism (ko00240), and linoleic acid metabolism (ko00591), among others.

### Melatonin regulated antioxidant enzyme activities and redox-related gene expression to protect melon roots from copper stress

**Fig. 4:**
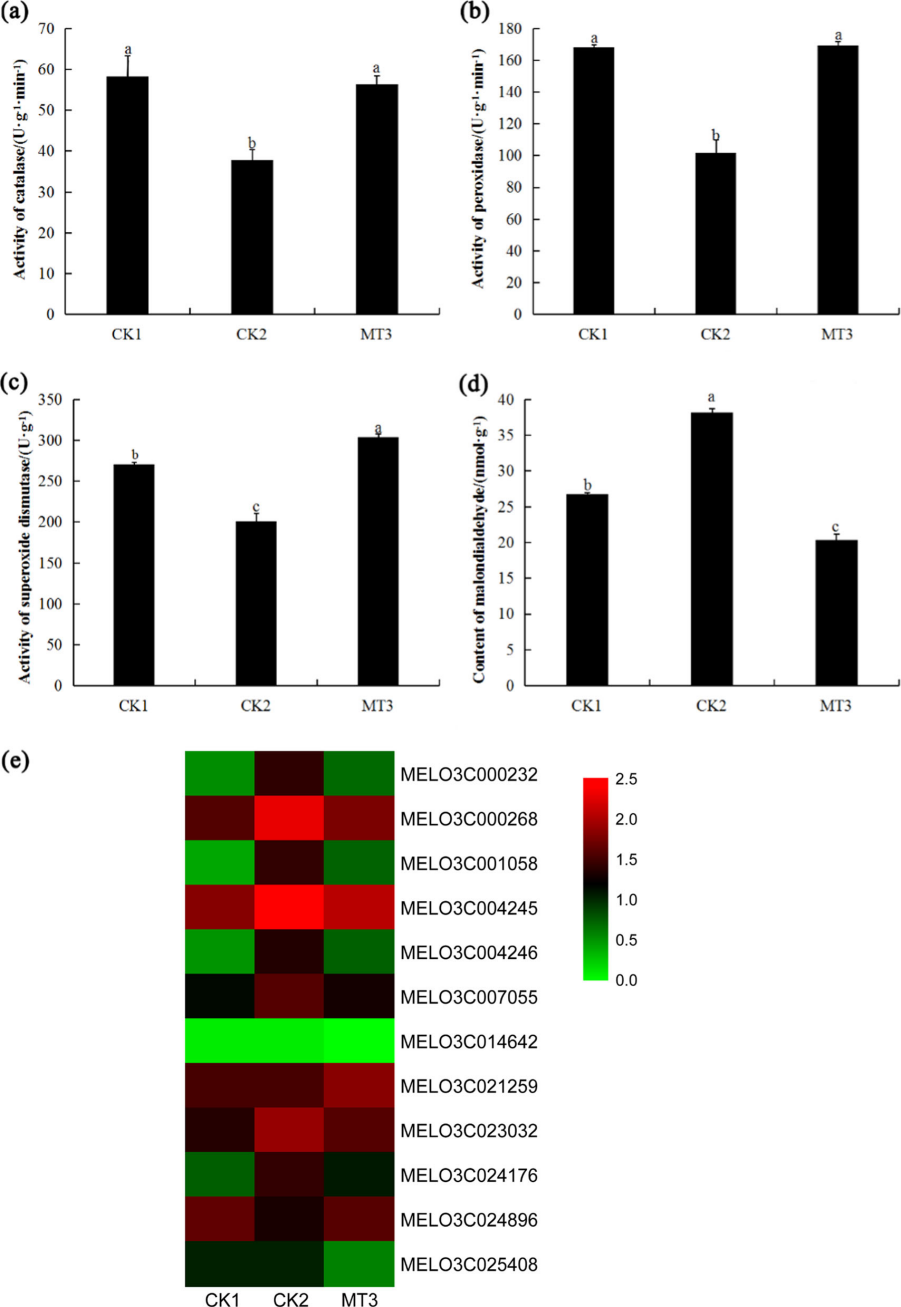
Effects of melatonin on redox enzymes and genes in melon. **a** Effect of melatonin on CAT activity. **b** Effect of melatonin on POD activity. **c** Effect of melatonin on SOD activity. **d** Effect of melatonin on MDA content in melon. **e** Effects of melatonin on the expression of redox genes in melon. CK1 non-stressed seeds pretreated with water, CK2 300 μmol/L CuSO_4_-stressed seeds pretreated with water, MT3 300 μmol/L CuSO_4_-stressed seeds pretreated with 100 μmol/L melatonin

Antioxidant enzymes have the ability to protect plants against oxidative damage induced by stress. In this study, we found that the CAT, POD and SOD activities of CK2 decreased by 35%, 39%, and 26% relative to those of CK1, respectively, but those of MT3 increased by 49%, 66%, and 51% relative to those of CK2, respectively (Fig. 4a–c). The MDA content exhibited the opposite trend; it increased by 40% in copper-stressed roots but decreased by 47% in melatonin-pretreated roots under copper stress (Fig. 4d). These results indicated that melatonin can alleviate the oxidative stress caused by excess copper in melon roots.

Many redox-related genes were detected, including *peroxidase*, *lipoxygenase* and *glutamate dehydrogenase*. Between CK1 and CK2, 51 genes were upregulated relative to those in CK1, and 26 genes were downregulated; however, between CK2 and MT3, 2 genes were upregulated relative to those in CK2, and 10 genes were downregulated (Supplementary Table S1). In total, nine genes that were assigned to the oxidation-reduction process (GO:0055114) were differentially expressed in all three treatments. Eight of these genes were upregulated by copper stress but downregulated by melatonin under copper stress, whereas one was downregulated by excess copper but upregulated by melatonin in Cu^2+^-stressed melon roots (Fig. 4e). Four *lipoxygenases* that were downregulated by melatonin under copper stress were also assigned to the membrane disassembly (GO:0030397) and jasmonic acid biosynthetic process (GO:0009695) functional groups (Supplementary Table S1).

### Melatonin altered cell wall-related gene expression to promote melon root formation

**Fig. 5:**
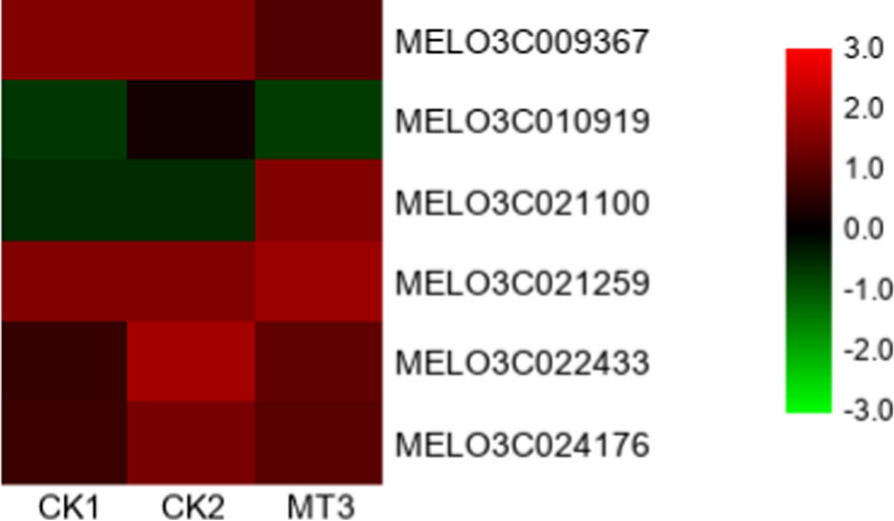
Effects of melatonin on the expression of cell wall-related genes in melon. CK1 non-stressed seeds pretreated with water, CK2 300 μmol/L CuSO_4_-stressed seeds pretreated with water, MT3 300 μmol/L CuSO_4_-stressed seeds pretreated with 100 μmol/L melatonin

Root development is closely related to cell wall formation. From the transcriptome analysis, we found that melatonin regulated the expression of cell wall-related genes. Between CK1 and CK2, there were 54 DEGs (13 genes upregulated and 41 genes downregulated relative to those in CK1), whereas between CK2 and MT3, there were 6 DEGs (2 genes upregulated and 4 genes downregulated relative to those in CK2). Melatonin pretreatment upregulated the expression of *peroxidase* and *heat shock 70* *kDa protein* but downregulated the expression of *xyloglucan endotransglucosylase/hydrolase (XTH)*, *dirigent protein*, *thaumatin-like protein*, and *subtilisin-like protease family protein*. The expression of genes encoding dirigent protein, thaumatin-like protein and subtilisin-like protease family protein showed the opposite trend in CK2 (Supplementary Table S1; Fig. 5). These findings suggested that melatonin influenced melon root development by regulating cell wall gene expression.

### Melatonin regulated the expression of transcription factor genes to influence root development

**Fig. 6:**
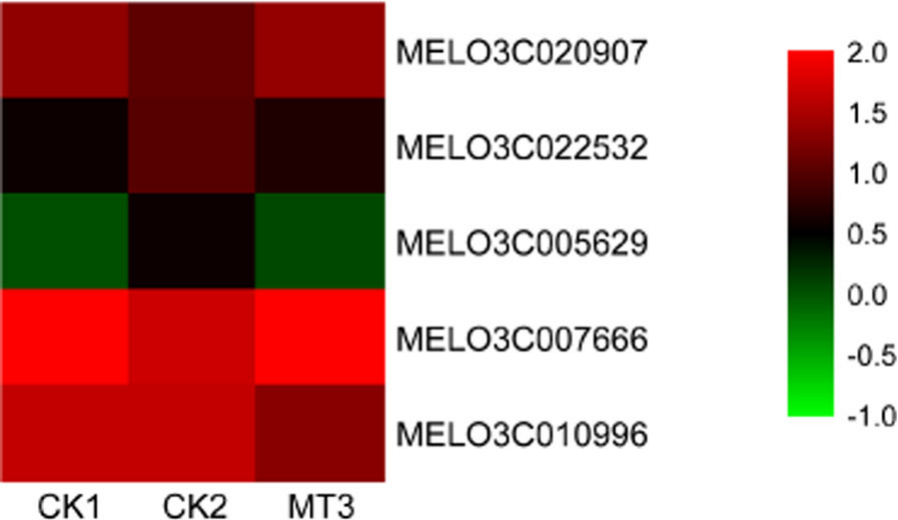
Effects of melatonin on the expression of transcription factor genes in melon. CK1 non-stressed seeds pretreated with water, CK2 300 μmol/L CuSO_4_-stressed seeds pretreated with water, MT3 300 μmol/L CuSO_4_-stressed seeds pretreated with 100 μmol/L melatonin

Transcription factors have a vital role in the regulation of genes in plants. We found 82 differentially expressed transcription factor genes under the copper treatment compared with the control treatment, including *MYB*s, *NAC*s, *WRKY*s, and *AP2/ERF*s. In addition, five transcription factor genes were differentially expressed between melatonin-pretreated and non-pretreated roots under copper stress, including *AP2/ERF*s, *BBR/BPC*s, *GRAS*s, and *HD-ZIP*s (Supplementary Table S5). In the melatonin-pretreated samples, *AP2/ERF*s and *BBR/BPC*s were significantly downregulated, whereas *GRAS*s were markedly upregulated. We found two significantly differentially expressed *HD-ZIP*s; one was upregulated by melatonin, whereas the other was downregulated. Four of these genes showed the opposite trend in CK2 (Fig. 6; Supplementary Table S5).

### Melatonin increased glutathione levels to promote copper chelation

GSH can chelate Cu^2+^ in cucumber under copper stress^24^. Our results showed that GSH exhibited different levels in different treatments. Compared with that in the CK1, the level of glutathione disulfide in the CK2 increased by a factor of 1.06. Compared with that in the CK2, the level of GSH in the MT3 increased by 28%. In this study, pretreatment with or without melatonin increased GSH levels in the roots of melon seedlings under copper stress (Supplementary Table S3).

### Melatonin regulated genes and metabolites involved in JA biosynthesis to promote melon root development

JA can inhibit plant roots by inhibiting root cell elongation, cell division^25^, and ROS damage^26^. Lipoxygenase (LOX) is the key enzyme involved in JA biosynthesis^27,28^. The major substrates for LOX are linoleic acid and linolenic acid^29^. A combined transcriptomic and metabolic analysis can better explain the transcriptional regulation of metabolic pathways. We simultaneously mapped differentially expressed genes and metabolites from the same treatment groups to KEGG pathways to clarify the relationships between genes and metabolites. Between CK2 and MT3, differentially expressed genes and metabolites were assigned only to linoleic acid metabolism (ko00591), which included four lipoxygenase-related genes (*MELO3C000232*, *MELO3C000268*, *MELO3C001058*, and *MELO3C004245*) and two metabolites (linoleic acid and lecithin), indicating that melatonin may have alleviated the effects of copper stress on roots by stimulating the lipoxygenase pathway (Supplementary Table S6). The four LOX-related genes were also enriched for linoleate the 13S-lipoxygenase activity (GO:0016165), linoleate 9S-lipoxygenase activity (GO:1990136), jasmonic acid biosynthetic process (GO:0009695), and membrane disassembly (GO:0030397) functional groups (Supplementary Table S1).

**Fig. 7:**
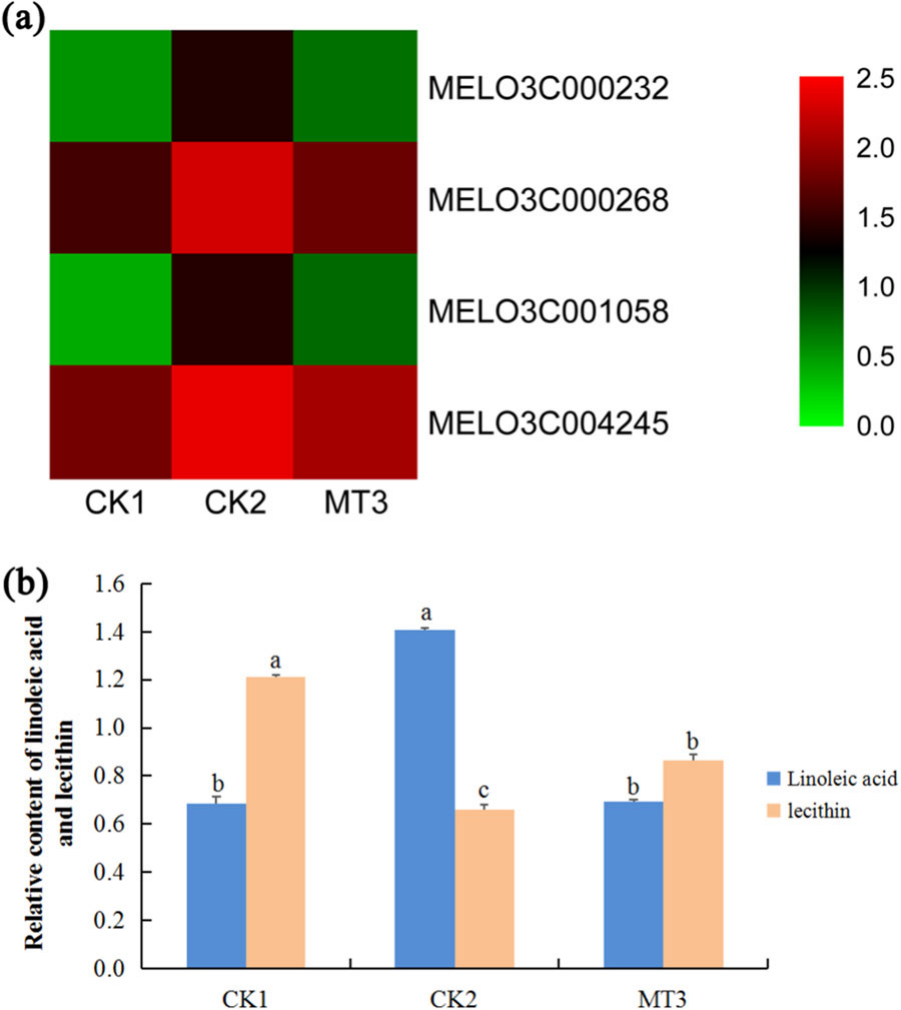
Effects of melatonin on genes and metabolites involved in JA biosynthesis in melon. **a** Hierarchical clustering of DEGs invovled in JA biosynthesis in melon. **b** The relative contents of linoleic acid and lecithin in melon. The relative concentration values increased 1000-fold in each treatment. CK1 non-stressed seeds pretreated with water, CK2 300 μmol/L CuSO_4_-stressed seeds pretreated with water, MT3 300 μmol/L CuSO_4_-stressed seeds pretreated with 100μmol/L melatonin

In the linoleic acid metabolism pathway, between CK1 and CK2, seven genes (*MELO3C000268*, *MELO3C001058*, *MELO3C004245*, *MELO3C014629*, *MELO3C000232*, *MELO3C014632*, and *Cucumis_melo_newGene_1656*) and linoleic acid levels were upregulated relative to those of CK1, whereas lecithin levels were downregulated (Supplementary Tables S1, S3, and S4). Compared with that in CK1, the expression of *MELO3C000268*, *MELO3C001058*, *MELO3C004245*, *MELO3C014629*, *MELO3C000232*, *MELO3C014632*, and *Cucumis_melo_newGene_1656* in CK2 increased by factors of 4.16, 15.42, 2.72, 1.91, 9.60, 2.41, and 8.32, respectively; at the same time, linoleic acid levels increased by 106%, whereas lecithin levels decreased by 45%. Between CK2 and MT3, four genes (*MELO3C000268*, *MELO3C001058*, *MELO3C004245*, and *MELO3C000232*) and linoleic acid were downregulated relative to those in CK2, whereas lecithin was upregulated (Fig. 7; Supplementary Tables S1, S3, and S4). Compared with that in the CK2, the expression of *MELO3C000268*, *MELO3C001058*, *MELO3C004245*, and *MELO3C000232* in the MT3 decreased by 69%, 83%, 53%, and 84%, respectively; at the same time, linoleic acid levels decreased by 51%, and lecithin levels increased by 31% (Fig. 7; Supplementary Tables S1 and S3). These results suggested that melatonin downregulated the level of linoleic acid and the expression of lipoxygenase-related genes, thus inhibiting the biosynthesis of JA.

## Discussion

As an important antioxidant and radical scavenger, melatonin can maintain the dynamic balance of free radicals and can reduce oxidative damage to plants caused by stress. Li et al.^5^ found that melatonin alleviated oxidative damage caused by salinity, perhaps by directly enhancing antioxidative enzyme activities or scavenging H_2_O_2_. Shi et al.^30^ found that exogenous melatonin improved the resistance of bermudagrass to drought, salt, and cold stress by enhancing antioxidative enzyme activities (i.e., POD, SOD, and CAT) and alleviating the reactive oxygen species burst. Compared with wild-type rice, melatonin-rich transgenic rice seedlings exhibited more resistance to oxidative stress induced by herbicides^31^. In this study, melatonin alleviated copper stress by regulating redox reactions in melon roots.

As shown in Figs. 1 and 2, copper toxicity decreased CAT, SOD, and POD activities; increased the MDA content; and inhibited lateral root development. Overall, these results indicated that copper harmed melon root systems. Similarly, copper stress can lead to changes in lipid peroxidation, antioxidant enzyme activities, and glutathione content in *Brassica juncea*^32^. Copper also affects root meristem cell proliferation, thus impacting maize root growth, and under excess copper conditions, plasmalemmas, mitochondrial membranes, and endoplasmic reticula were disturbed^33^. In contrast, melatonin-pretreated samples exhibited better melon lateral root development; higher activities of CAT, SOD, and POD; and a lower MDA content. These results indicated that melatonin pretreatment obviously alleviated the inhibition of melon lateral root development caused by copper stress. Thus, melatonin had a positive effect on melon root development in a concentration-dependent manner under CuSO_4_ stress, and the MT3 treatment best alleviated copper toxicity. This experiment demonstrated that root development of melon under CuSO_4_-stress was promoted by a low concentration of melatonin and inhibited by a high concentration of melatonin, as has been similarly observed with auxin, in agreement with research by Sarropoulou et al.^23^

We detected 12 redox-related genes with significant differential expression between the CK2 and MT3 treatments. One of them is a peroxidase-encoding gene that was upregulated in the MT3 treatment, reflecting the peroxidase activity observed in the roots. Peroxidases are present in almost all plant tissues and are particularly abundant in roots^34^; as important plant enzymes, peroxidases are involved in many physiological processes^35^. *AtPrx33* and *AtPrx34*, two highly homologous *Arabidopsis* peroxidase-encoding genes, are involved in cell elongation. *AtPrx34*-overexpressing seedlings had significantly longer roots, whereas seedlings with reduced *AtPrx33* or *AtPrx34* expression had shorter roots^36^. Exogenous melatonin pretreatment upregulated peroxidase-related genes and increased peroxidase activity in cucumber roots, thus promoting lateral root formation in cucumber plants under salt stress^37^. Four LOX genes were downregulated by melatonin in this study. LOXs catalyze the oxygenation of polyunsaturated fatty acids, including linoleic acid, and degrade lipid bilayers and cytomembranes^38^. In addition, superoxide anion, peroxide, and free radicals generated by LOXs are cytotoxic and capable of damaging membranes, proteins, and DNA^39^. Researchers have detected the upregulation of LOX transcript levels in various plant species under different forms of stress, including wounding, cold, desiccation, and salt stress^40–43^. Melatonin alleviated oxidative damage by increasing antioxidant enzyme activities, decreasing MDA levels and regulating redox gene expression.

In plants, LOX is involved in polyunsaturated fatty acid metabolism and is the key enzyme involved in lipid degradation and JA biosynthesis^27,28^. Lecithinase, with lecithin as a substrate, catalyzes the biosynthesis of linoleic acid, and LOX and other enzymes catalyze the conversion of linoleic acid to synthesize JA^28^. Leaves of transgenic *Arabidopsis* that lacked LOX2 accumulated less JA than did control plants in response to wounding^44^. JA is an endogenous growth-regulating substance in plants, with a vital role in plant growth^45^, and acts as a signaling molecule involved in stress responses^46,47^. Under stress, the accumulation of JA largely increases; for example, tobacco infected with *Pseudomonas syringae* pv. *phaseolicola* showed increased JA accumulation and *13-LOX* expression in leaves within 3–9 h^48^. In addition, studies have shown that the restraint of plant root growth is controlled by JA. JA has been shown to exert a repressive effect on *Arabidopsis* primary root growth^49^. JA inhibited rice roots by inhibiting root cell elongation, cell division^25^, and ROS damage^26^. In our study, the combined comparative transcriptomic and metabolic analysis between CK2 and MT3 revealed that four LOX-related genes and linoleic acid levels were downregulated in MT3, whereas lecithin levels were upregulated. Melatonin pretreatment inhibited linoleic acid biosynthesis and downregulated *LOX* expression, thus controlling the JA content in the roots of melon seedlings under copper stress. Melatonin promoted melon lateral root development under copper stress by reducing both damage to LOX and the inhibition of JA.

The expression of cell wall-related genes was closely associated with melon lateral root formation. Melatonin regulated cell wall formation, thus affecting lateral root development. Between CK2 and MT3, six cell wall-related genes were significantly differentially expressed: *peroxidase*, *xyloglucan endotransglucosylase/hydrolase* (*XTH*), *heat shock 70* *kDa protein*, *dirigent protein*, *thaumatin-like protein* and *subtilisin-like protease family protein*. *Peroxidase* and *heat shock 70* *kDa* protein were significantly upregulated in the MT3 treatment, whereas the others were significantly downregulated. XTHs have an important role in cell wall remodeling and expansion, with *XTH* expression changing under stress. The transcript level of maize *wusl1005* [*gfu*], which encodes a homolog of xyloglucan endotransglycosylase, was enhanced under flooding stress^50^. In *Sagittaria pygmaea*, anoxia increased the expression of *SpXTH1* and *SpXTH4*^51^. Similarly, at 24 h after *Arabidopsis* roots were treated with aluminum, the expression level of *XTH-5* exhibited a statistically significant increase^52^. According to another study, two XTH proteins affected *Arabidopsis* root growth; compared with the controls, *Arabidopsis* seedlings incubated with AtXTH14 and AtXTH26 showed shorter and fewer root hairs^53^. Our results showed that the transcript level of *XTH* was obviously decreased by melatonin pretreatment. In addition, *XTH* expression was upregulated under copper stress, and *XTH* overexpression may inhibit melon root development. It has been shown that activity of the heat shock 70 kDa protein can lead to a large increase in root formation^54^. In our study, the expression of *heat shock 70* *kDa protein* was decreased in CK2 but increased in MT3. Thus, melatonin pretreatment can alleviate copper stress and promote melon root development by downregulating *XTH* expression and upregulating *heat shock 70* *kDa protein* expression.

Transcription factors, such as activators, inhibitors, or both, control the expression of genes and influence many aspects of plant growth and development. We found that in melatonin-pretreated samples, *AP2/ERF*s and *BBR/BPC*s were significantly downregulated, whereas *GRAS*s were markedly upregulated. We identified two significantly differentially expressed *HD-ZIP* transcription factors: one was upregulated by melatonin, whereas the other was downregulated. *AP2/ERF*s have been shown to be involved in responses to multiple abiotic stresses, including drought, salt, and heat stresses^55^. Rice *OsDRE1F* overexpression can increase tolerance to salt, drought, and low temperatures in both rice and *Arabidopsis*^56^. *BBR/BPC*s are related to root development, and accordingly, the number of lateral roots and lateral root primordia was reduced in *Arabidopsis bpc1-1 bpc2 bpc4 bpc6* quadruple mutants, whereas no phenotypic changes were observed in single-gene-mutants^57^. Our results indicated that melatonin might alleviate the toxicity of copper and promote melon root development and, accordingly, reduce the expression of *AP2/ERF* and *BBR/BPC* transcription factor genes. SCARECROW is a GRAS family member, and in *Arabidopsis AtSCR* mutants, roots developed aberrantly^58^. The *SCARECROW-like* gene (*CsSCL1*) isolated from chestnut has been shown to have an essential role in the initial process of adventitious root formation^59^. In the present research, *GRAS* overexpression induced by melatonin may promote the development of melon roots. *GLABRA2* (*GL2*) belongs to the class IV HD-ZIP family, and *GL2* is necessary for the regulation of root hair development in *A. thaliana*^60,61^. *ATHB6*, an HD-ZIP gene, was significantly upregulated in *A. thaliana* seedlings under osmotic and water deficit stress and in response to exogenous abscisic acid (ABA) treatment^62^. Similar to *ATHB6*, both *ATHB7* and *ATHB12* were upregulated under water deficit^63^. In cucumber, *CsHDZI12* was repressed under high-salinity conditions, whereas *CsHDZI4* was repressed by low temperature^64^. In the present study, melatonin regulated the expression of HD-ZIP-related genes to promote root growth and development. These results suggest that transcription factors have an essential role in root development induced by melatonin.

GSH exists in reduced glutathione and oxidized glutathione forms and is involved in both the sequestration of heavy metals and the detoxification of ROS^65^. GSH can chelate excess metals in plants. In cadmium-exposed oat roots, GSH was found to be associated with cadmium^66^. In cucumber, GSH chelated excess Cu^2+^ and formed heavy metal complexes that were transported to the vacuole^24^. In our study, we found that with and without melatonin pretreatment increased the level of GSH in copper-stressed melon roots. Compared with that in CK1, the GSH level in CK2 increased, which may reflect melon’s defensive reaction. Melatonin pretreatment further increased the GSH level in MT3, which allowed the melon roots to develop better than those in CK2.

**Fig. 8:**
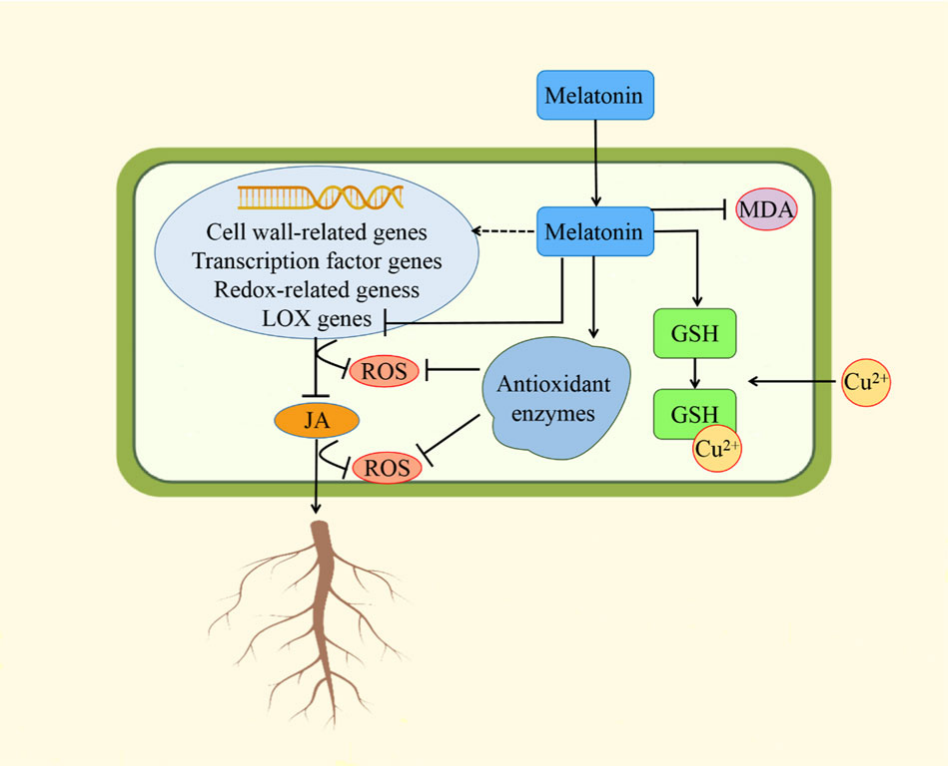
Schematic diagram of the mechanism by which melatonin alleviates copper toxicity

Our results suggested that melatonin could promote melon root development by regulating linoleic acid metabolism. Melatonin decreased the level of linoleic acid and the expression of four LOX-related genes, thus decreasing the JA level. Melatonin decreased ROS damage by reducing LOX-related gene expression and JA levels, regulating the expression of other redox genes and increasing antioxidant enzyme activities. Melatonin also altered the expression of genes related to cell wall formation processes and of members of the AP2/ERF, BBR/BPC, GRAS, and HD-ZIP transcription factor families. In addition, melatonin increased the level of GSH, which was chelated excess Cu^2+^. In conclusion, melatonin alleviated copper toxicity and promoted melon root development via multiple mechanisms (Fig. 8).

## Materials and methods

### Plant materials

Plump melon seeds were allotted to one of eight treatments. The seeds of treatments MT1– MT6 were pretreated in melatonin solutions (concentration of 10, 50, 100, 300, 500, or 800 μmol/L, respectively) at 25 °C for 12 h. Seeds in the CK1 and CK2 treatments were pretreated in water at 25 °C for 12 h. Afterwards, the CK2 and MT1–MT6 seeds were germinated on three layers of filter paper moistened with a 300 μmol/L CuSO_4_ solution, whereas the CK1 seeds were germinated on three layers of water-moistened filter paper. The seeds were then cultivated at 30 °C in darkness for 3 days. Each treatment was replicated five times, with 1000 seeds each. The roots were separated from seedlings and used as materials for additional experiments and analysis.

### Measurement of antioxidant enzyme activities, proline content, malondialdehyde content, and root vigor

In accordance the methods of Li et al.^67^ we measured antioxidant enzyme activities, proline content, MDA content, and root vigor. POD, CAT, and SOD activities were measured by guaiacol, spectrophotometry, and nitro blue tetrazolium methods, respectively. Proline and MDA contents were measured using the ninhydrin reaction and thiobarbituric acid colorimetric methods, respectively. Last, the 2,3,5-triphenyltetrazolium chloride (TTC) method was used to measure root vigor.

### RNA quantification and qualification

RNA was extracted from melon root tissue, with three replicates per treatment. Total RNA was extracted using a Uniq-10 column TRIzol total RNA extraction kit. We measured RNA concentrations using a NanoDrop 2000 (Thermo Fisher Scientific, Waltham, MA, USA) and assessed the RNA integrity using a RNA Nano 6000 Assay Kit in conjunction with an Agilent Bioanalyzer 2100 system (Agilent Technologies, Santa Clara, CA, USA).

### Library construction for sequencing

RNA sample sequencing was conducted using 1 μg of RNA per sample. We constructed sequencing libraries using a NEBNext Ultra RNA Library Prep Kit for Illumina (New England Biolabs, Ipswich, MA, USA) and added index codes to facilitate attributing sequences to samples. For each sample, mRNA was removed from total RNA using poly-T oligo-attached magnetic beads. Dissociation was performed using divalent cations under elevated temperatures. First-strand cDNA synthesis was performed using random hexamer primers and M-MuLV Reverse Transcriptase, and second-strand cDNA synthesis used DNA Polymerase I and RNase H. The remaining overhangs were converted into blunt ends using polymerase or exonuclease activities. DNA fragments with polyadenylation at their 3′ ends were connected to NEBNext adaptors with a hairpin loop structure to prepare for hybridization. The fragments were purified using an AMPure XP system (Beckman Coulter, Beverly, MA, USA) to select cDNA fragments of 240 bp in length. Adaptor-ligated and size-selected cDNA was and 3 μL of USER Enzyme (New England Biolabs) were then mixed together at 37 °C for 15 min, after which the mixture was incubated at 95 °C for 5 min before PCR. PCR was performed with Index (X) Primer, Universal PCR primers, and Phusion High-Fidelity DNA polymerase. Last, we purified PCR products using an AMPure XP system and assessed library quality using an Agilent Bioanalyzer 2100 system.

### Clustering and sequencing

The clustering of the index-coded samples was conducted using a cBot Cluster Generation System with a TruSeq PE Cluster Kit v4-cBot-HS (Illumina, San Diego, CA, USA) following the manufacturer’s recommendations. The prepared libraries were sequenced on an Illumina platform, and paired-end reads were produced after cluster generation.

### Transcriptomic data analysis

Clean reads were produced by removing low-quality reads as well as reads containing adaptor sequences or poly-N sequences from raw reads. Moreover, the sequence duplication level, GC content, and percentages of Q20 and Q30 reads were calculated from clean reads. High-quality clean reads were used for downstream analyses.

The clean reads were mapped to the reference genome sequence, and reads that were perfectly matched or contained one mismatch were further analyzed and annotated on the basis of the reference genome. We used HISAT2 tools to map the reads to the reference genome. Gene expression levels were estimated as fragments per kilobase of transcript per million fragments mapped (FPKM).

Differential expression analyses among the three treatments (CK1 vs. CK2, CK1 vs. MT3, CK2 vs. MT3, with three biological replicates per treatment) were conducted using the DESeq R package (1.10.1). DESeq uses a statistical model based on a negative binomial distribution to identify differentially expressed genes from digital gene expression data. Benjamini and Hochberg’s approach was used to adjust *p*-values, and the FDR was controlled for by adjusting the *p*-value threshold. The fold change indicates the ratio of expression between the two treatments within a comparison. Genes with a fold change ≥ 1.5 and an FDR < 0.05 were considered to be DEGs.

GO is a set of annotation categories pertaining to the biological processes, molecular functions, and cellular components of various genes. GO enrichment analysis of the DEGs was performed using the GOseq R package^68^. The KEGG^69^ database is a resource that systematizes the high-level functions of biological systems according to biological pathways (http://www.genome.jp/kegg/). KOBAS^70^ software was used to test the statistical enrichment of DEGs in the KEGG pathways.

### Quantitative real-time PCR validation

Q-PCR was used to validate the RNA-seq data for 12 different genes. Specific primers were designed using Primer Premier 5 software (Premier Biosoft, Palo Alto, CA, USA) (Supplementary Table S7). The RNA samples were used to synthesize cDNA, and a StepOnePlus Real-Time Fluorescent Quantitative PCR system (ABI, Foster City, CA, USA) was used to monitor the amount of DNA. Assays of each gene were repeated three times. Quantification was evaluated using the 2^−(∆∆Ct)^ method.

### Extraction and quantification of metabolites

Metabolites were extracted from melon root tissue, with five replicates per treatment. Metabolites were analyzed using an Agilent 1290 ultra-high-performance liquid phase chromatograph. Together with Analyst TF 1.7 software, an AB5600 Triple TOF mass spectrometer in IDA mode was used to collect primary and secondary mass spectral data. Molecular ions with the strongest intensity and exceeding 100 were then chosen to acquire the corresponding secondary mass spectral data during each data acquisition cycle. The energy was 30 eV in the electron impact mode, with 15 secondary mass spectra/50 ms. The ESI ion source parameters were as follows: the atomization pressure (GS1) was 60 psi, auxiliary pressure was 60 psi, air curtain pressure was 35 psi, temperature was 650 °C, and spray voltage was −4000 V (negative ion mode) or 5000 V (positive ion mode).

The raw mass spectral data were converted into mzXML format using ProteoWizard Toolkit^71^. XCMS was then used for retention time correction, peak identification, peak extraction, peak integration, peak alignment, and so on. Afterward, we used an in-house R package and a custom-made secondary mass spectrometry database to identify peaks. The peak area data of a single peak or for all groups with a null value no more than 50% was retained after a single peak was filtered. The missing value in the original data was filled using the minimum value 1/2 simulated method. The total ion current (TIC) of each sample was used for normalization.

### Metabolomic data analysis

We used a combination of the *p*-values of Student’s *t*-tests with the VIP value of the OPLS-DA model to filter differentially expressed metabolites, with additional screening criteria (*p* < 0.1, VIP > 0.1). We constructed metabolic pathways based on the information in the KEGG database.

### Combined metabolomic and transcriptomic analysis

The metabolomic and transcriptomic data were converted to log2 values before analysis. We used Pearson correlation coefficients (PCCs) and corresponding *p*-values (PCCPs) to screen metabolites and related genes within the combined metabolomic and transcriptomic analysis; the screening criteria were PCC > 0.80 and PCCP < 0.05. To better understand the relationship between genes and metabolites, we mapped the differentially expressed genes and metabolites among the same treatments (CK1, CK2, and MT3) to their associated KEGG pathways.

## Supplementary information

Table S1

Table S2

Table S3

Table S4

Table S5

Table S6

Table S7
